# Neuraxial Anesthesia for Combined Left Nephrectomy and Left Hemicolectomy in a One-Lung Patient

**DOI:** 10.7759/cureus.59854

**Published:** 2024-05-07

**Authors:** Davide Vailati, Emilio Bonvecchio, Gianmarco Secco, Carmelo Magistro, Benedetta Basta

**Affiliations:** 1 Anesthesia and Intensive Care, Melegnano Hospital-ASST Melegnano e Martesana, Vizzolo Predabissi, ITA; 2 General Surgery, Melegnano Hospital-ASST Melegnano e Martesana, Vizzolo Predabissi, ITA

**Keywords:** neuraxial anesthesia, thoracic spinal anesthesia, one-lung patient, frailty patient, open abdominal surgery, awake anesthesia

## Abstract

Monopulmonary patients undergoing major abdominal surgery represent a high-risk population. While general anesthesia is typically the standard approach, mechanical ventilation can cause significant complications, particularly in patients with pre-existing lung conditions. Tailored anesthesia strategies are essential to mitigate these risks and preserve respiratory function. We present the case of a 71-year-old female with a history of prior right pneumonectomy for lung cancer. She was scheduled for combined left nephrectomy and left hemicolectomy laparotomic surgery because of extended colon cancer. The patient was prepared according to the local Enhanced Recovery After Surgery (ERAS) protocol and underwent thoracic neuraxial anesthesia with sedation maintaining spontaneous breathing, so avoiding general anesthesia and mechanical ventilation. Anesthesia in the surgical field was effective, and no respiratory problems occurred intraoperatively. The patient's rapid recovery and early discharge underscore the success of our "tailored anesthesia strategy." Our experience highlights the feasibility and benefits of tailored anesthesia in monopulmonary patients undergoing major abdominal surgery. By avoiding general anesthesia and mechanical ventilation, we mitigated risks and optimized patient outcomes, emphasizing the importance of individualized approaches in high-risk surgical populations.

## Introduction

A monopulmonary patient requiring major abdominal surgery is by definition at high risk [1]. The anesthesiological approach must therefore consider mitigating the risks associated with the proposed anesthesia. While general anesthesia appears to be the gold standard in major abdominal surgery, it is also true that mechanical ventilation can lead to mechanical, functional, and infectious complications in some individuals. Particularly in patients with pre-existing lung diseases, and even more so in monopulmonary patients, the target of a "tailored anesthesia" is even more crucial, aiming to protect respiratory function in all its forms: reducing the risk of respiratory failure and infectious complications in the postoperative period. Therefore, opting for an alternative strategy to general anesthesia and mechanical ventilation could represent the possibility of achieving the desired outcome; in this regard, we considered thoracic neuraxial anesthesia with the maintenance of spontaneous breathing [2,3].

## Case presentation

We describe the case of a 71-year-old female patient with a history of hypertension, significant smoking history (30 cigarettes/day for more than 30 years), and previous right pneumonectomy for lung cancer, who was diagnosed with left colon cancer with left kidney infiltration. This necessitated a combined left nephrectomy and left hemicolectomy. The patient was prepared according to the Enhanced Recovery After Surgery (ERAS) protocol in the 14 days preceding the surgery [4]. To minimize the impact of surgery on the patient's lung function, we opted for an "awake anesthesia" approach, using thoracic neuraxial anesthesia with intraoperative sedation while maintaining spontaneous breathing.

Given the need to achieve visceral and somatic coverage for the kidney and left colon removal (targeting T10-L1 for the kidney and left colon and T12-S4 for the rectosigmoid) [5], we chose to perform a combined thoracic spinal-epidural anesthesia at the T7-T8 level using levobupivacaine 0.5% 8.5 mg+dexmedetomidine 5 mcg (total volume 4 ml) and a saddle block anesthesia with bupivacaine 0.5% 10 mg+dexmedetomidine 5 mcg (total volume 2.2 ml). Following the thoracic spinal anesthesia, when hypotension occurred, a norepinephrine infusion was initiated for the first 30 minutes. Intravenous sedation was maintained using dexmedetomidine infusion at a variable dosage of 0.2-1 mcg/kg/hour, along with ketamine boluses of 10-20 mg during surgically sensitive moments (total 100 mg). Throughout the procedure, sedation was maintained at a Richmond Agitation-Sedation Scale (RASS) score of -2/-1, analyzing electroencephalographic waveform using Bispectral Index™ monitoring system monitoring (BIS™) [6]. Hemodynamics and fluid status were guided using pro-AQT™ (Pulsion Medical Systems, Munich, Germany, called hereafter PulsioFlex™) monitoring after radial artery cannulation. Spontaneous ventilation was maintained throughout the procedure, with normocapnic arterial blood gases and a P/F ratio >300 (baseline value 322; see Table 1).

**Table 1:** ABG sample during surgery and recovery room admission ABG: arterial blood gas; T0: ABG before anesthesia (in spontaneous breathing); T1: ABG after one hour surgery; T2: ABG after two hours surgery; T3: ABG after three hours surgery; recovery room: ABG after recovery room admission (FiO2 33%)

ABG	Reference values	T0	T1	T2	T3	Recovery room
pH	7.35-7.45	7.33	7.34	7.34	7.41	7.36
pCO2 (mmHg)	35-45	41	40.7	38.3	31	38
pO2 (mmHg)	80-100	106	106	126	147	179
HCO3- (mmol/L)	21-28	21	21.5	20	21.1	21
BE (mmol/L)	-2/+2	-4.4	-4	-5	-4.2	-3.4
Hb (g/dL)	12-16	12	12.2	13.4	13.7	13.3
pO2/FiO2	>350	322	322	382	448	540
AnGap (mmol/L)	8-18	12.5	12.7	14	12.8	8.8
Lac (mmol/L)	0.5-2.2	1.19	1.3	1.23	1.6	0.8

The patient was kept in spontaneous breathing using a high-flow nasal cannula (HFNC) system (FiO2 40%, flow 45 liters/min, temperature 37°C). Sensory block effectiveness was monitored using the Nociception Level (NOL™) index (PMD-200 system from Medasense; Ramat Gan, Israel), which remained constant throughout the procedure [7]. The epidural catheter was used only for postoperative analgesia (starting at the end of surgery), through the administration of ropivacaine 0.2% (2 mg/ml) at a dosage between 4 and 6 ml/hour.

## Discussion

Even if general anesthesia represents the standard of care in performing major abdominal surgery, neuraxial anesthesia could be considered as an alternative, especially in high-risk populations. Spinal thoracic puncture is able to produce segmental anesthesia, which means anesthesia only of metameric levels involved in surgery. Potential advantages of thoracic segmental anesthesia include avoidance of mechanical ventilation and related complications and preservation of neurological function because of the absence of pharmacological GABAergic neurological effects [8].

We decided to perform a combined thoracic spinal-epidural anesthesia to obtain anesthesia (with the spinal injection) and to manage postoperative analgesia or possible prolongation of surgical times beyond the duration of spinal anesthesia (with an epidural catheter). We also performed a "saddle block anesthesia" (a kind of low spinal block that manifests anesthesia over the saddle area, i.e., the perineum and perianal area) for the perineal part of the surgery, guaranteeing correct coverage of the pain resulting from that specific surgical maneuver. The choice of levobupivacaine as a mild hypobaric anesthetic aimed to utilize its stability and cranial spread to somatic and visceral levels beyond the puncture site, ensuring control of pain and discomfort from intra-abdominal manipulation [9]. Dexmedetomidine was used as an adjuvant to increase the block's duration and extension [10]. The patient's written consent was obtained regarding its off-label use. Planning a four-hour procedure duration, we also placed an epidural catheter to extend anesthesia duration if needed, along with ensuring optimal postoperative analgesia and patient mobilization.

The sedation regimen aimed to minimize the impact of GABAergic drugs on neurological function and the risk of perioperative delirium, utilizing dexmedetomidine's qualities [11]. BIS-guided EEG monitoring helped achieve a sedation level that maintained effective breathing without causing emotional sequelae or intraoperative recall [12]. Ketamine contributed to maintaining patient immobility during critical surgical phases due to its dissociative properties.

Maintaining spontaneous breathing preserved diaphragmatic function, unaffected by thoracic spinal anesthesia, as evidenced by the absence of postoperative lung atelectasis. A pre- and postoperative lung ultrasound was followed: in both cases, the Lung Ultrasound Score (LUS) was 0 [13]. Noradrenaline was chosen for hemodynamic control to counter sympathetic block effects, targeting a MAP >70mmHg to ensure effective cerebral, coronary, and splanchnic circulation and proper perfusion of surgical anastomoses.

Muscle relaxation of the abdominal wall was optimal, facilitating correct surgical progression without impact from spontaneous ventilation. The patient's rapid systemic functional recovery led to early discharge without neurocognitive and respiratory sequelae.

## Conclusions

The neuraxial approach with thoracic spinal anesthesia, combined with controlled sedation avoiding GABAergic drugs, successfully preserved the patient's remaining lung function using spontaneous breathing. Dexmedetomidine in the subarachnoid space effectively maintained spinal anesthesia, achieving excellent pain control. The successful outcome and high satisfaction from both surgeon and patient suggest the potential of thoracic spinal anesthesia as a "tailored approach," highlighting its advantages over conventional methods based solely on "medical traditions."
